# Effects of organic fertilizers on growth characteristics and fruit quality in Pear-jujube in the Loess Plateau

**DOI:** 10.1038/s41598-022-17342-5

**Published:** 2022-08-04

**Authors:** Shenglan Ye, Biao Peng, Tiancheng Liu

**Affiliations:** 1 Institute of Land Engineering and Technology, Shaanxi Provincial Land Engineering Construction Group Co., Ltd., Xi’an, 710075 China; 2 Shaanxi Provincial Land Engineering Construction Group Co., Ltd., Xi’an, 710075 China; 3 Key Laboratory of Degraded and Unused Land Consolidation Engineering, Ministry of Natural Resources, Xi’an, 710075 China

**Keywords:** Plant sciences, Ecology, Environmental sciences

## Abstract

The ecological environment of the hilly and gully area of the Loess Plateau in northern Shaanxi is fragile and the soil fertility is low. As a result, the yield and quality of Pear-jujube which constitute one of the dominant economic forests in this region, have been severely restricted. At present, the scientific application of fertilizer is important for comprehensively improving the quality of fruit trees, and for devising the optimal management of fruit trees. In particular, the application of organic fertilizers plays an important role in improving soil and improving fruit quality. In this experiment, a field study was conducted to understand the effects of different organic fertilizer applications on physiological growth, photosynthetic characteristics, reproductive growth and nutritional quality of Pear-jujube in the Loess Plateau. The results showed that organic fertilizer significantly promoted the physiological growth of Pear-jujube. The Pear-jujube bearing branch and leaf area under the soybean cake fertilizer (SC) treatment were 20.17 cm and 1246 mm^2^/leaf, respectively, which are increased by 34% and 44.46% compared with the no fertilizer treatment, which was a control check (CK). The total chlorophyll content of fertilization treatment was significantly higher than that of CK (*P* < 0.05). The maximum of chlorophyll content was 10.90 mg/dm^2^ under the biogas fertilizer (BM). The content of LAI was in the order BM > conventional fertilizer (CF) > sheep manure (SM) > SC > CK. The changing trend of gap fraction was opposite to that of LAI, and the density of light was consistent with that of LAI. The density of light BM was the largest, reached 38.06 mol/(m^2^ d), which was 15.13% higher than that of CK. Organic fertilizer significantly improved the net photosynthetic rate (Pn) and water use efficiency (WUEp) of Pear-jujube. The WUEp of SC was up to 3.30%. Organic fertilizer significantly promoted the reproductive growth and improved the nutritional quality of Pear-jujube. The yield under the SC was 19,177 kg/hm^2^, increased by 138.5% compared with that of CK. The fruit water content (FWC), total soluble solids (TSS), solid-acid ratio (TSS/TA), Vc and total flavonoids content improved, and the maxima of FWC, TSS, TSS/TA, Vc and total flavonoids content under the SC treatment were 86.30%, 18.48%, 40.17, 46.18 mg/kg and 14.35 mg/kg, respectively, which were significantly different from those of CK (*P* < 0.05). Organic fertilization effectively promotes the growth, development, yield and fruit quality of Pear-jujube in the Loess Plateau and the effect of the soybean cake fertilizer is the most significant.

## Introduction

The ecological environment in the hilly and gully regions of the Loess Plateau in northern Shaanxi is fragile. Soil fertility is low. To protect the environment and make use of land resources, a large number of economic forests are planted in this area to reconvert farmlands to forests. This can effectively green the barren hills, conserve water and soil, and improve the ecological environment. This can also increase the economic income of farmers. Pear-jujube is a rare and precious variety of jujube. It has rich nutritional value. At present, it has become one of the leading plant varieties in the economic forest industry in northern Shaanxi^1^. The long-term and sustainable development of the Pear-jujube Forest is inseparable from a good water-and-fertilizer environment. However, there is a problem with the long-term application of quick-acting chemical fertilizers and pesticides in the production of Pear-jujube. This will not only cause serious environmental pollution but also affect the nutritional quality of Pear-jujube^2,3^. Chen et al.^4^ studied the effect of different nitrogen, phosphorus and potassium fertilizers on the yield and fruit quality of jujube, and concluded that the highest yield fertilizer ratio was 1:0.84:0.11. And it is proposed that under the same fertilizer base conditions, the total acidity of red dates is positively correlated with the nitrogen fertilizer application amount, and the reducing sugar and vitamin C content and nitrogen and phosphorus fertilizer application amounts show a trend that is first increasing and then decreasing. Excessive application or partial application of chemical fertilizer is to exchange high input for high yield; although the yield problem was solved, it also caused a series of environmental effects such as groundwater pollution and serious soil degradation^5,6^. Moreover, excessive application of chemical fertilizers will also have adverse effects on the metabolism of organic compounds in plants^7^. Because some fruit farmers only pay attention to the application of chemical fertilizers with less or no use of organic fertilizers, problems such as reduced soil organic matter, acidification, nutrient imbalance, and serious diseases in orchards are caused^8^. The application of those highly toxic and high residue pesticides increase the toxic components of grains, vegetables and fruits. Food safety is not guaranteed, thus endangering human health. In recent years, with the accompanying increase in the number of chemical fertilizers and crop yields, the number of organic fertilizers has also increased^9^.

At present, the fertilization system and technology in organic planting in china are not mature and perfect. Therefore, studies on how to reasonably use field management measures in organic planting, and appropriately regulates the types of fertilizers are needed. Scientific fertilization and soil fertilization are very necessary. The application of organic fertilizer can not only improve soil fertility^10,11^, improve soil physical and chemical properties^12,13^, and enhance soil water storage capacity^14,15^ but also effectively promote the vegetative growth and reproductive growth of plants, thereby improving the quality of plants^16–19^. The experimental results of Yu et al.^20^ showed that organic fertilizer can promote rice to reduce ineffective tillers, increase panicle rate, increase effective panicle number and late green leaf area, increase leaf-grain ratio, and improve rice seed setting rate and grain filling fullness. This increases the yield effect of increased panicle weight. The application of different organic fertilizers can significantly reduce the nitrate content of peppers^21^. Wang et al.^22^ found that the yield of jujubes with medium and high dosages of organic fertilizers without biochar addition was the highest, reaching 39.49 and 40.16 kg per plant. The application of a high proportion of organic fertilizer and biochar could not significantly improve the photosynthetic capacity of jujube; on the contrary, the treatment of a low application amount of organic fertilizer without biochar could significantly improve the photosynthetic capacity of jujube. Biochar has high adsorption. Excessive application of biochar increased soil porosity, thereby reducing soil effective porosity. The movement of nutrients and water to the root system of crops is affected. It is not conducive to the absorption of nutrients and water, thereby reducing the photosynthetic capacity of jujube. This shows that the combined application of organic fertilizer and biochar is not the best fertilization mode for jujube in the short term. Zaituniguli et al.^23^ researched that long-term fertilization can increase the chlorophyll content in crop leaves. At the grain filling stage, the SPAD value of the NP treatment was the lowest, which was 37.07; the SPAD value of the NPK + organic fertilizer treatment was the highest, reaching 44.62, and the difference was significant (*P* < 0.05). The biological yield of NPK + organic fertilizer treatment was the highest, equaling 94.81 t/hm^2^, which was 97.95% higher than that of CK. Therefore, research on the application of organic fertilizer and photosynthesis is key in exploring the yield and quality of Pear-jujube.

With the rapid growth of the industrial economy in the Loess Plateau region of northern Shaanxi and the acceleration of the construction of energy and chemical bases, the layout, production structure and product structure of the agricultural industry can be further optimized. Taking advantage of the huge reserves of farmyard manure in the Loess Plateau, it can supply and develop characteristic organic agriculture and become an effective way to increase farmers' income. At present, there are few research reports on the effects of applying organic fertilizers on the photosynthetic physiological characteristics and water use of Pear-jujube in the hilly and gully regions of the Loess Plateau. For this reason, this study takes Pear-jujube in the loess hilly area as the research object, and applies different organic fertilizers during the flowering and fruit setting period. We also analyze the comprehensive effects of organic fertilizer on the vegetative growth, reproductive growth, photosynthetic characteristics and water use of Pear-jujube. The research further explores the organic fertilizer source suitable for the growth and development of Pear-jujube. This can provide a theoretical basis and technical support for the production of organic Pear-jujube.

## Materials and methods

### Overview of the test area

The experiment was carried out at the Pear-jujube micro-irrigation technology experimental demonstration base (37.78′ N, 110.23′ E, 870 m above sea level) in Mizhi County, Yulin City, Shaanxi Province from 2018 to 2019. This area is a typical hilly and gully area of the Loess Plateau, with a mid-temperate semi-arid climate, with an average annual temperature of 8.5 °C and an average annual rainfall of 451.6 mm, mainly from July to September. The main soil in the test area is Lossiah soil. The physical and chemical properties of the soil layer at 0–20 cm before fertilization are as follows: the available nitrogen, phosphorus, and potassium contents are 34.73, 2.90, and 101.9 mg/kg, respectively, and the organic matter content is 2.1 g/kg and pH 8.6, bulk density of 1.21 g/cm^3^ and the soil particle composition is as shown in Table 1. The entire slope of the orchard has a height difference of approximately 100 m. The terrain is complex, and the slope directions are different. During the trial period, the drip irrigation quota was 135 m^3^/hm^2^, and irrigation was carried out on May 15th and June 10th, respectively.

**Table 1:** Soil particle composition.

Layers (cm)	Sand (%) (1–0.05 mm)	Silt particle (%) (0.05–0.001 mm)	Clay (%) (< 0.001 mm)	Physical clay (%) (< 0.01 mm)
0–20	28.6	67.9	2.01	17.4
20–40	28.1	68.0	2.38	16.3
40–60	32.2	65.3	2.31	15.8

### Experimental design

We selected the 7-year-old mountain dwarf and densely planted Pear-jujube (*Zizyphus jujuba* Mill.cv.) with a uniform tree body and good growth. We set up five fertilization treatments under drip irrigation conditions, namely: no fertilizer (CK); conventional fertilizer^24^ (CF), urea (containing N 46%) 0.48 kg/plant, superphosphate (containing P_2_O_5_ 12%) 1.35 kg/plant, potassium sulfate (containing 50% K_2_O) 0.61 kg/plant; fermented and decomposed soybean cake fertilizer (SC), 5 kg per plant; decomposed sheep manure (SM), 15 kg/plant; and biogas fertilizer (BM), 100 kg per plant. The application rate is calculated based on the content of each nutrient in the organic fertilizer and based on the consistent application of nitrogen, phosphorus, and potassium. A single plant is regarded as one treatment, and a total of five repetitions are set. The test area was 300 m^2^, and the planting density was 2 m × 3 m. The soybean cake fertilizer used in the test is the oil residue obtained after oil extraction. It is sealed and fermented for 35 days under the condition of relative humidity of 70%. It was then used as a decomposed soybean cake fertilizer. The mixture of biogas slurry and biogas residue, decomposed sheep manure is a mixture of crushed straw and sheep manure buried in a water pit and subjected to microbial anaerobic fermentation. The nutrient content of each organic fertilizer is shown in Table 2.

**Table 2:** Nutrient content of the experimental organic fertilizers.

Organic fertilizer	Moisture content (%)	Organic matter (%)	Total N (%)	Total P (%)	Total K (%)
Soybean cake fertilizer	22.74	7.69	4.13	0.91	1.57
Sheep manure fertilizer	8.23	24.72	0.95	0.73	2.25
Biogas manure fertilizer	99.43	0.17	0.21	0.096	0.11

### Measurement indicators and methods

Organic fertilizer was applied in early April 2019. On May 31, June 6, June 14, June 22, and July 1, 2019, 12 jujube hangings were selected from the upper parts of the plant in four directions: east, west, south and north. The jujube hangings are in good shape and uniform in growth. The leaf area and leaf relative water content of the 5th leaf from the top were determined. In mid-June, the leaf chlorophyll content, photosynthetic index, canopy index and soil water content (SWC) were measured, and the number of flowering plants was investigated. In mid-July the number of fruits and was counted and the fruit set rate was calculated. On October 8, the ripe fruits were collected from each tree to determine the quality.

The length of the jujube hanging was measured with steel tape; 12 jujube hangings were fixedly selected in different directions on the upper part of each plant, and the length was measured regularly with a steel tape measure.

The leaf area was measured with a portable leaf area meter model AM300 (ADC-UK). The leaf area was periodically measured on the fifth leaf of the fixed jujube.

The relative water content (RWC) of the leaf was measured using the saturation weighing method. The fresh weight (Wf) of the leaves was first weighed. Then the leaves were immersed in distilled water for 6–8 h, so that the leaves were saturated with water. After the leaves were taken out, surface water was wiped with absorbent paper, and immediately put into a weighing bottle of known weight to weigh. Then the leaf was immersed into distilled water for a while, taken out and dried outside, and then weighed until the weight no longer increases. This is the saturated water weight (Wt) of the leaf. The samples were then dried at 70 °C at constant weight to obtain tissue dry weight (Wd). The calculation formula is RWC = (Wf − Wd)/(Wt − Wd) × 100%.

The chlorophyll content of leaves was measured using the acetone method, and the content of chlorophyll a, b and the total amount of chlorophyll were calculated^25^. Fresh leaves with thick veins were cut into pieces, and extracted with 80% acetone in the dark. The extract was taken and measured for colorimetry using a 722 type visible light spectrophotometer at the absorbance wavelengths of 663, 645 and 652 nm, and 80% acetone was used as a reference.

The photosynthetic indexes were measured using the LI-6400XT photosynthesis instrument (Li-COR, USA). The red and blue light sources that come with the leaf chamber were used. The intensity of photosynthetically active radiation was set to 2500 μmol/(m^2^ s), with an open gas path. The temperature was 25 °C. The CO_2_ concentration was 400 μmol/mol. Pn, Ci, Gs and Tr of leaves were determined after Pear-jujube matured, with five replicates per plant.

WUEp was calculated using Pn and Tr when the photosynthetically active radiation was 2500 μmol/(m^2^ s). The formula was WUEp = Pn/Tr.

The measured Pn, Gs, Ci and Tr were compared according to the values when the photosynthetically active radiation was 2500 μmol/(m^2^ s).

Canopy indicators were determinedusing WinsCanopy2005a canopy analysis. Image acquisition was performed in the field. Then, canopy analysis and supporting analysis software was used to process the images. The leaf area index (LAI), forest gap fraction (GFR), total radiant flux above the canopy and total radiant flux below the canopy were measured, and the transmittance and the density of light were calculated.

Transmittance = total average density of photosynthetically effective radiation below the canopy/total average density of photosynthetically effective radiation above the canopy.

The density of light = the average density of total photosynthetically effective radiation above the canopy—the average density of total photosynthetically effective radiation below the canopy^26^.

SWC was measured using conventional soil drilling and drying method. The sampling location was 30 cm away from the tree body, the measuring depth was 0–100 cm soil layer, and the sampling interval was 20 cm.

Determination of fruit quality: The water content of the fruit was measured using the normal pressure heating and drying method^27^; the total soluble solids (TSS) were measured using the 2WAJ-Abbe refractometer; the titratable acid (TA) was measured using the 0.1 mol/L NaOH standard solution Titration method^28^; reduced vitamin C was titrated with 2,6-dichloroindophenol^29^; and total flavonoids were determined using NaNO_2_–Al(NO_3_)_3_ spectrophotometry^30,31^.

The average value of each treatment was taken as the final result. The experimental data was organized in Microsoft Excel 2003. Data analysis of variance was performed using DPS 7.05 software to test significant differences between data processing.

## Results and analysis

### Effect of different organic fertilizers on the growth of Pear-jujube

#### Effect of different organic fertilizers on the bearing branch length of Pear-jujube

**Figure 1:**
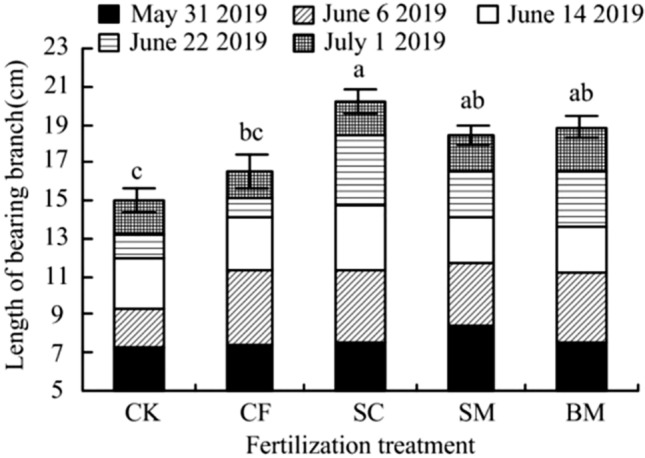
Effects of different fertilizer treatments on bearing a branch of Pear-jujube. *Note*: At different levels, each population mean follows a normal distribution with the same variance. The least significant difference (LSD) method was selected for analysis of variance in DPS software. Different lowercase letters indicate significant difference between treatments (*P* < 0.05).

Jujube-bearing branch has the dual role of fruiting and photosynthesis^32,33^. It can be seen from Fig. 1 that different organic fertilizer treatments have a significant impact on the growth of jujube-bearing branches. Among them, the longest jujube-bearing branch in the SC treatment is 20.17 cm, which is significantly higher than that in CK and CF; the jujube-bearing branch length in the SC, SM and BM treatment are increased by 34%, 23% and 25% compared with that in CK, and the difference is significant (*P* < 0.05), compared with CF treatment, the increase is 22%, 12%, and 14%, respectively. Starting from May 31st, the length of the jujube-bearing branch in each treatment increased significantly. By the end of June, the length of the jujube-bearing branch in the CK and CF treatments grew slowly, while the length of the jujube-bearing branch in the SC, SM and BM treatments increased by 3.76 cm, 2.53 cm, and 2.96 cm respectively.

#### Effect of different organic fertilizers on the leaf area of Pear-jujube

**Figure 2:**
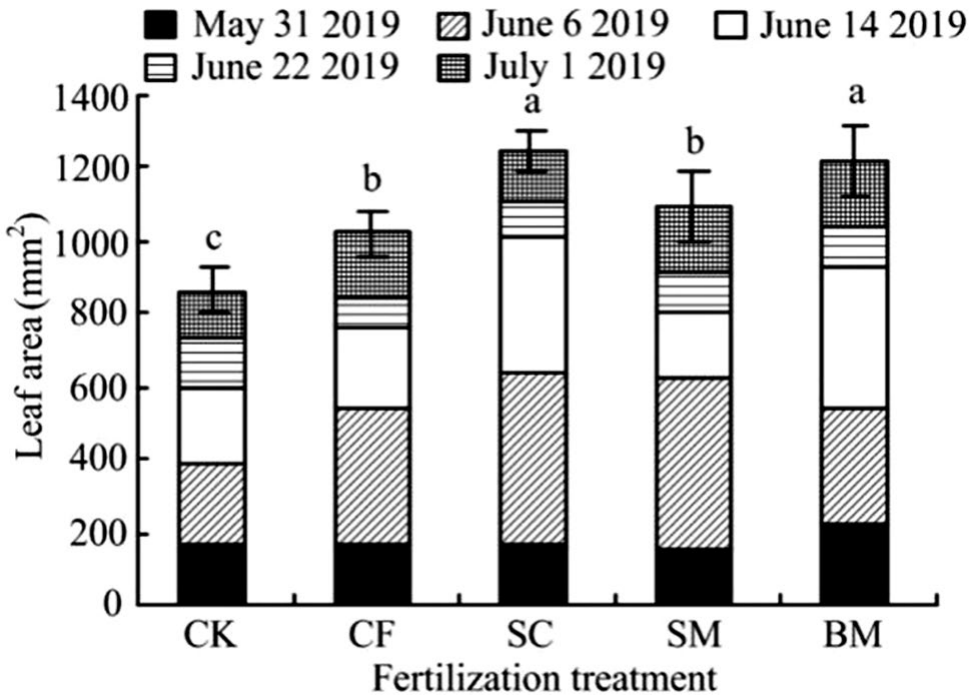
Effects of different fertilizer treatments on leaf area of Pear-jujube. *Note*: At different levels, each population mean follows a normal distribution with the same variance. The least significant difference (LSD) method was selected for analysis of variance in DPS software. Different lowercase letters indicate a significant difference between treatments (*P* < 0.05).

Figure 2 shows that the increase in leaf area of each fertilization treatment is significantly different from that of the CK (*P* < 0.05). The leaf area of the SC treatment is the largest, with a leaf area of 1246 mm^2^/leaf. The leaf area of the SC and BM treatments are significantly different from that of the CK, CF and SM treatments (*P* < 0.05); Compared with that of CK, the leaf area of CF, SC, SM and BM treatments increased by 18.34%, 44.46%, 26.67%, and 41.65%. Compared with CF treatment, SC, SM, and BM organic fertilizer treatments increased by 22.08%, 7.04%, and 19.70%, respectively. Soybean cake fertilizer (SC) and biogas fertilizer (BM) saw the most significant increases.

### Effect of organic fertilizers on the photosynthetic effect of Pear-jujube during the flowering and fruit setting period

#### Effect of organic fertilizers on the chlorophyll content of Pear-jujube during flowering and fruit setting

The content of chlorophyll affects the absorption and conversion of light energy. The change in the ratio of chlorophyll a and chlorophyll b (chl a/b) can reflect the strength of leaf photosynthetic activity and the amount of light energy used by plants^34^. The photosynthetic performance of Pear-jujube during the flowering and fruit setting period directly affects the supply of nutrients required for the flowering and fruit setting of Pear-jujube, which is of great significance to the final yield. It can be seen from Table 3 that four treatments have different effects on the chlorophyll content of Shandi Pear-jujube during the flowering and fruit setting period. The total chlorophyll content is significantly higher than that of CK. CF, SC, SM and BM increased by 22.86%, 26.73%, 39.31% and 43.01%, respectively, comparing with CK. The content of chlorophyll a is in the order SM > BM > SC > CF > CK. It is found that organic fertilizer can significantly improve the chlorophyll a content. The changing trend of chlorophyll b content is consistent with the total content. The analysis found that the chl a/b content of CK is the highest. The chl a/b content of BM, SC and CF are significantly less than CK. It may be due to fertilizing that the synthesis of chlorophyll b was greater than that of chlorophyll a. This can significantly increase the absorption of the plants to blue-green light, enhance the photosynthetic activity of the blade. Among them, the application of biogas fertilizer has the most significant effect on the chlorophyll content, and the total chlorophyll content is 10.90 mg/dm^2^, while chl a/b is only 3.27.

**Table 3:** Effects of different fertilizer treatments on chlorophyll contents of Pear-jujube.

Treatment	Chl a (mg/dm^2^)	Chl b (mg/dm^2^)	TC (mg/dm^2^)	Chl a/b
CK	6.08 ± 0.04 c	1.54 ± 0.06 c	7.62 ± 0.10 d	3.97 ± 0.13 a
CF	6.97 ± 0.09 b	2.10 ± 0.02 b	9.36 ± 0.34 c	3.61 ± 0.01 b
SC	7.76 ± 0.19 a	2.11 ± 0.15 b	9.66 ± 0.04 bc	3.60 ± 0.30 b
SM	8.38 ± 0.21 a	2.33 ± 0.01 ab	10.61 ± 0.34 ab	3.82 ± 0.05 a
BM	8.34 ± 0.61 a	2.55 ± 0.17 a	10.90 ± 0.78 a	3.27 ± 0.03 c

The data are the mean ± standard deviation of the parameters in this group. At different levels, each population mean follows a normal distribution with the same variance. The least significant difference (LSD) method was selected for analysis of variance in DPS software. Different lowercase letters in the same column indicate significant difference between treatments (*P* < 0.05).

*TC* Total chlorophyll.

#### Effects of organic fertilizers on the canopy structure and canopy optical properties of Pear-jujube during flowering and fruit setting

**Figure 3:**
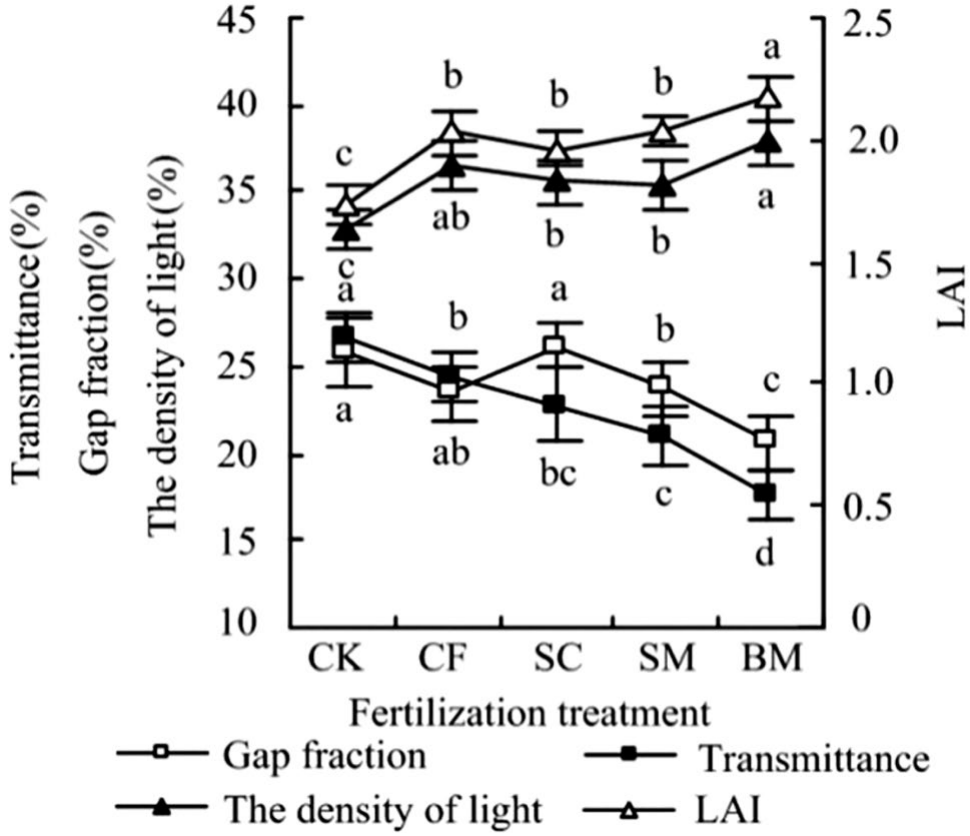
Effects of different fertilizer treatments on canopy characteristics of Pear-jujube. *Note*: Note: At different levels, each population mean follows a normal distribution with the same variance. The least significant difference (LSD) method was selected for analysis of variance in DPS software. Different lowercase letters indicate a significant difference between treatments (*P* < 0.05).

It can be seen from Fig. 3 that LAI of Pear-jujube after fertilization is significantly higher than that of CK. Compared with CK, CF, SC, SM, and BM increases by 15.75%, 12.46%, 15.62%, and 24.50%, respectively. The LAI of BM is up to 2.17, and it is significantly different from other treatments (*P* < 0.05). The changing trend of gap fraction in different treatments is opposite to that of LAI. Canopy photosynthetically active radiation is the most important indicator for evaluating canopy transmittance and light interception ability. The density of light of different treatments is consistent with LAI, which is expressed as BM > CF > SM > SC > CK. Among them, the density of light of BM is the largest. It reaches 38.06 mol/(m^2^ d). CF, SC, SM and BM respectively increase by 11.54%, 8.09%, 7.96% and 15.13% compared with CK, and the difference is significant. The canopy transmittance of jujube is BM < SM < SC < CF < CK, which are reduced by 8.64%, 14.78%, 21.62% and 34.16% compared with CK, respectively. The organic fertilizer treatments are significantly different from CK (*P* < 0.05).

#### Effects of organic fertilizers on photosynthetic characteristics and leaf water use efficiency of Pear-jujube during flowering and fruit setting

The organic fertilizer significantly increases the Pn and Gs of Pear-jujube. The Pn and Gs in each treatment are in the order BM > SM > SC > CF > CK. The Pn and Gs of BM are the highest, which are 22.38 µmol/(m^2^ s) and 0.5014 mmol/(m^2^ s), respectively. After fertilization, the Ci is less than that of CK. It shows that fertilization effectively reduce Ci. Among them, the effect of BM which is only 240.8 µmol/mol is the most obvious. Transpiration is the same as photosynthesis; they are regulated by many factors. Stomatal transpiration is the main method of transpiration^35,36^. The Tr of different treatments is expressed in the order BM > CK > CF > SM > SC. The highest Tr of BM reaches 8.66 µmol/moL. It may be related to higher LAI, and the instantaneous water use efficiency of SC is highest, which reaches 3.30%. The WUEp of CF, SC, SM and BM treatments increase by 22.4%, 64.2%, 44.3% and 30.8%, respectively, compared with that of CK. It reaches a significant difference level (*P* < 0.05). The instantaneous water use efficiency of SC, SM and BM is 0.84, 0.44 and 0.17 percentage points higher than that of CF, respectively (Table 4).

**Table 4:** Effects of different organic fertilizers on photosynthetic characters of Pear-jujube.

Treatment	Pn (µmol/(m^2^ s))	Gs (mmol/(m^2^ s))	Ci (µmol/mol)	Tr (µmol/mol)	WUEp (%)
CK	18.30 ± 1.87 c	0.4011 ± 0.0304 c	270.2 ± 3.16 a	8.54 ± 0.31 a	2.01 ± 0.12 d
CF	20.80 ± 1.61 b	0.4547 ± 0.0456 b	255.3 ± 4.67 bc	8.30 ± 0.45 a	2.46 ± 0.17 c
SC	22.19 ± 0.90 a	0.4912 ± 0.0320 a	252.0 ± 4.11 c	6.57 ± 0.33 c	3.30 ± 0.26 a
SM	22.37 ± 0.49 a	0.4991 ± 0.0352 a	255.9 ± 3.57 b	7.67 ± 1.06 b	2.90 ± 0.19 b
BM	22.38 ± 0.97 a	0.5014 ± 0.0329 a	240.8 ± 3.74 d	8.66 ± 0.20 a	2.63 ± 0.13 bc

Pn-Net photosynthetic rate, Gs-Stomatal Conductance, Ci-Intercellular CO_2_ concentration, Tr-transpiration rate, WUEp—water use efficiency. The data are the mean ± standard deviation of the parameters in this group. At different levels, each population mean follows a normal distribution with the same variance. The least significant difference (LSD) method was selected for analysis of variance in DPS software. Different lowercase letters in the same column indicate significant difference between treatments (*P* < 0.05).

### Effect of organic fertilizers on water utilization during flowering and fruit setting period of Pear-jujube

#### Effect of organic fertilizers on SWC during flowering and fruit setting of Shandi Pear-jujube

**Figure 4:**
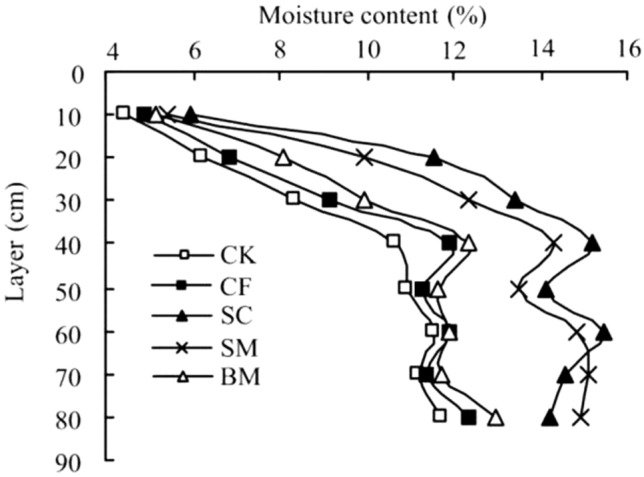
Effects of different fertilizer treatments on soil water content.

The SWC of each treatment increases rapidly with the increase of soil depth and then tends to be gentle. Among them, the SWC of SC and SM is significantly higher than that of BM, CF and CK. This is because the application of organic fertilizer can make the soil absorb a lot of water and prevent the infiltration of water. The average SWC of each treatment in the 0–80 cm soil layer is SC > SM > BM > CF > CK. Compared with CK (9.37%), the SC, SM, BM, and CF increased by 3.69, 3.18, 1.11 and 0.40% points, respectively. Organic fertilizer is beneficial to increase the water content of the soil. Among them, soybean cake fertilizer (SC) has the largest increase, which is significantly different from CK (*P* < 0.05) (Fig. 4).

#### Effect of organic fertilizers on relative water content during flowering and fruit setting of Pear-jujube

**Figure 5:**
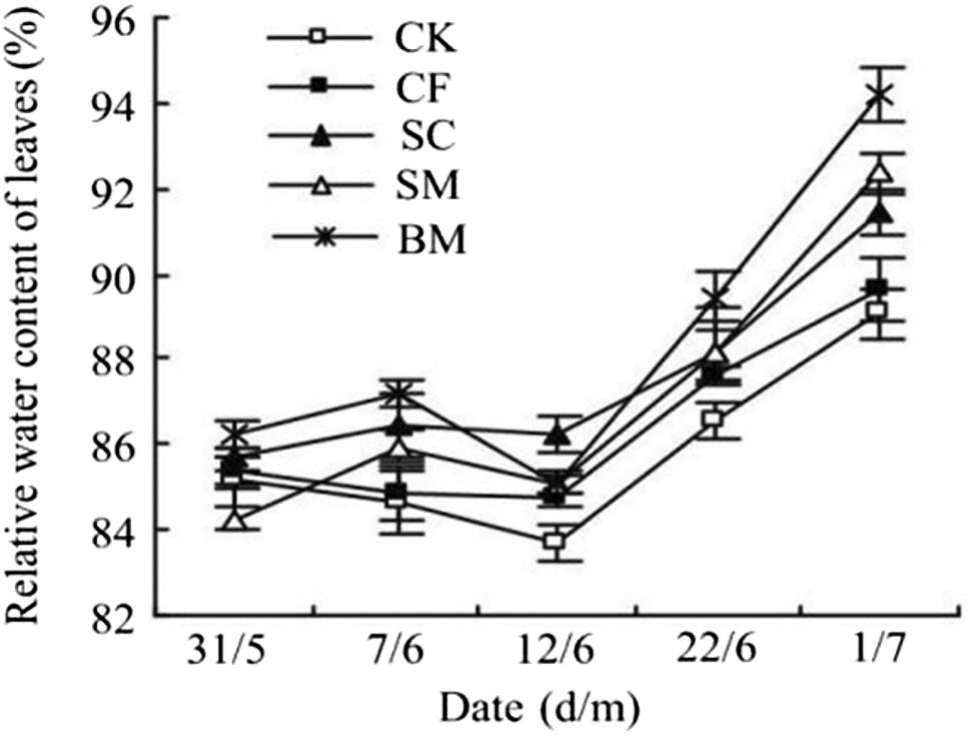
Effects of different fertilizer treatments on relative leaf water content of Pear-jujube.

The flowering period requires sufficient humidity, so the high relative water content (RWC) helps to set up flowers and improve the photosynthesis of Pear-jujube. It can be seen from Fig. 5 that the trend of leaves RWC changes of Pear-jujube in each treatment is a slow rise. It decreases slightly on June 12. This may be related to the large demand for water of jujube trees at this time, in which the application of organic fertilizer can improve the RWC of Pear-jujube leaves, thus supplying the water required for flowering. By July 1, the RWC of the leaves reaches the highest. The RWC for each treatment is in the order BM > SM > SC > CF > CK. The RWC of BM reaches 94.20%, which is significantly different from CK (*P* < 0.05). It can be seen that the application of organic fertilizer can improve the RWC of leaves. The RWC of BM, SM and SC increase by 5%, 3% and 2%, respectively, compared with that of CF.

### Effect of different organic fertilizers on the reproductive growth of mountain Pear-jujube

It can be seen from Table 5 that the total flowers, fruit number and yield per plant after fertilization are all greater than those of CK, and SC treatment has the most significant effect on reproductive growth (*P* < 0.05). Compared with CK, the total flower number of CF, SC, SM and BM increases by 42.59%, 82.68%, 14.48% and 40.39%, respectively, and the fruit yield per plant increased by 95.47%, 157.27%, 68.79% and 148.48%, respectively. It shows that different fertilization treatments significantly increase the fruit setting rate of Pear-jujube. The highest fruit setting rate of BM is 4.57%. The fruit setting rate and yield of each fertilization treatment are significantly higher than CK. Different organic fertilizers have varying degrees of influence on the reproductive growth of Pear-jujube. In combination with the above indicators, SC and BM have the most significant effects.

**Table 5:** Effects of different fertilizer treatments on reproductive growth of Pear-jujube.

Treatment	Flower No. (No./plant)	Fruit No. (No./plant)	Fruit setting rate (%)	Yield (kg/hm^2^)
CK	14,790 ± 1061 c	383 ± 19 c	2.54 ± 0.20 c	8040 ± 568 d
CF	21,564 ± 1660 b	748 ± 81 b	3.50 ± 0.65 b	11,797 ± 741 c
SC	27,629 ± 4552 a	985 ± 121 a	3.58 ± 0.20 b	19,177 ± 836 a
SM	18,246 ± 2044 bc	646 ± 51 b	3.58 ± 0.58 b	12,025 ± 891 c
BM	21,232 ± 5072 bc	951 ± 132 a	4.57 ± 0.61 a	14,142 ± 812 b

The data are the mean ± standard deviation of the parameters in this group. At different levels, each population mean follows a normal distribution with the same variance. The LSD least significant difference method was selected for analysis of variance in DPS software. Different lowercase letters in the same column indicate significant difference between treatments (*P* < 0.05).

### Effect of different organic fertilizers on the nutritional quality of Pear-jujube

Table 6 shows that the fruit water content of SC and BM increases by 9.15 and 8.26 percentage points, respectively, compared with that of CK, and it reaches a significant difference (*P* < 0.05). TSS, solid-to-acid ratio (TSS/TA), Vc and total flavonoid content of organic fertilizer treatments are higher than those of CK. The TSS of all treatments except SM is significantly different than that of CK (*P* < 0.05). The titratable acid (TA) content is in the order CK > SM > BM > SC, and the lowest TA of SC is 0.23% points lower than that of CK. The Vc content of SC is the highest, reaching 46.18 mg/kg. It is an increase of 78.92% compared with CK. The Vc content of BM and SM also increase by 59.59% and 43.36%, respectively, compared with those of CK. The total flavonoid content of each fertilization treatment is SC > SM > BM > CK. The total flavonoid content of SC reaches 14.35 mg/kg, which is 24.57% higher than that of CK. The total flavonoid content of SM and BM increase by 17.01% and 9.2%, respectively, compared with that of CK. Moreover, each treatment is significantly different from CK (*P* < 0.05). There is no significant difference in the total flavonoids content between SM and BM.

**Table 6:** Effects of different fertilizer treatments on nutrient quality of Pear-jujube.

Treatment	FWC (%)	TSS (%)	TA (%)	TSS/TA	Vc (mg/kg)	Total flavones (mg/kg)
CK	77.15 b	15.45 c	0.69 a	22.39 c	25.81 c	11.52 c
SC	86.30 a	18.48 a	0.46 c	40.17 a	46.18 a	14.35 a
SM	76.32 b	16.63 bc	0.53 b	31.38 b	37.00 b	13.48 b
BM	85.41 a	17.05 b	0.51 b	33.43 b	41.19 b	12.58 b

At different levels, each population mean follows a normal distribution with the same variance. The least significant difference (LSD) method was selected for analysis of variance in DPS software. Different lowercase letters in the same column indicate significant difference between treatments (*P* < 0.05).

*FWC* Fruit water content, *TSS* Total soluble solid, *TA* Titratable acid.

## Discussions

The flowering and fruit setting periods of Pear-jujube are lasts for a long time. This overlapping period shows the coexistence of vegetative growth and reproductive growth. It is more sensitive to nutrition and water conditions. Adequacy of its nutrient supply is directly related to the growth and yield of jujube. Therefore, scientific fertilization and soil cultivation fat are very necessary. Organic fertilizer can activate the nutrients in the substrate, improve the physical and chemical properties of the soil, promote the absorption of nutrients by plants, increase the nutrient content^37^, provide the nutrients needed for dry matter accumulation, and promote vegetative growth and reproductive growth^13,38^.

A study by Xue et al.^39^ has shown that the proportion of leaves and short branches in the seaweed fertilizer treatment was the highest (59.53%), while the proportion of long branches and leggy branches was the lowest (33.23%), and the leaf area reached 35.91 cm^2^ which was significantly higher than that of the control. The results of this study showed that the new shoots and leaf areas of SC, SM, and BM were significantly different from those of CK (*P* < 0.05) after the application of organic fertilizers, which increased by 34%, 23%, 25% and 44.46%, 26.67%, and 41.65%b compared with that of CK.

Chlorophyll is the main pigment for plant photosynthesis, and it plays a central role in light absorption in photosynthesis^40^. A study by Wang et al.^41^ has shown that after applying organic fertilizer, the chlorophyll content of wheat leaves is the highest, and SPAD value may reach up to 60.1, which is 58% higher than that of CK. A study by Li^42^ has shown that organic fertilizers from different plant sources increased the Pn of crisp jujube leaves to varying degrees and adjusted the transpiration rate of the leaves. This is consistent with the results of this research. The total chlorophyll content in the flowering and fruit setting period was in the order BM > SM > SC > CF > CK. The Pn indicates the accumulation of photosynthetic products in plants, and increasing the photosynthetic efficiency can produce more organic matter, which is beneficial to plant growth and development. The changing trend of Pn and Gs was consistent with the change in chlorophyll content. This may be because different organic fertilizers improve soil fertility^43^, which in turn enhances the photosynthesis of leaves and makes them accumulate more photosynthetic products. The intercellular CO_2_ concentration was opposite to the changing trend of leaf photosynthetic rate and stomatal conductance. It may be that non-stomata factors reduce the utilization of CO_2_ and cause the accumulation of CO_2_^44–46^. Therefore, the application of organic fertilizer significantly increases the chlorophyll content of the Pear-jujube during the flowering and fruit setting period, enhanced the intensity of photosynthesis, caused a decrease in the intercellular CO_2_ concentration, and accelerated the synthesis and accumulation of photosynthetic products.

A study by Chen et al.^47^ has shown that organic fertilization can significantly increase the rhizosphere moisture content and reduce the loss rate of soil water. In this study, the rhizosphere moisture content with organic fertilizer was significantly increased, especially in the depth of 40–80 cm in the soil layer of the widest distribution of absorbing roots. The SWC of SC and SM was significantly higher than that of CK and CF. This may be related to the strong water retention effect of organic fertilizers. At the same time, it also increased the relative water content of the leaves at the flowering stage. The relative water content of the leaves of BM was the highest (94.20%). Compared with CF, it increased by 5%. Organic fertilizer significantly improveds the water use efficiency of Pear-jujube. This may be due to the application of organic fertilizer being able to improve the total soil porosity and nutrient status^48,49^, which is beneficial to plant growth and water use. Although BM has the highest relative water content, its transpiration rate is also large, which reduces its water use efficiency. The SWC of SM and SC are both high, but the SC has the highest water use efficiency, Therefore, the application of SC can significantly improve the water utilization of Pear-jujube, and effectively retain water; this can meet the water demand of Pear-jujube during the flowering period.

The application of organic fertilizer improves the vegetative growth and water utilization of Pear-jujube. This is conducive to the accumulation of dry matter during the flowering period, and provides sufficient nutrition for reproductive growth to improve quality. In the organic fertilizer treatment, the fruit setting rate of the application of biogas fertilizer is the highest. This may be because the Pear-jujube itself has less flowering and requires relatively fewer nutrients. However, the Pear-jujube treated by the soybean cake fertilizer has too much flower volume, and in the case that the number of fruits did not reach a significant difference, the fruit setting rate was relatively low. The yield of Pear-jujube after applying organic fertilizer was significantly different from that of CK (*P* < 0.05). Among them, soybean cake fertilizer had the best effect, which is138.52% higher than CK. Not only the yield affects the sales of Pear-jujube, but also the water content and TSS/TA of the fruit directly affect the sales of Pear-jujube. A study by Zang et al.^43^ has showns that the application of organic fertilizer can increase the content of soluble solids and Vc in jujube fruit, and significantly reduce the content of titratable acid. After applying organic fertilizer, the water content of the fruit was significantly increased, and the difference between SC and CK treatments was significant (*P* < 0.05). TSS, TSS/TA, Vc and total flavonoids content were all higher than those of CK, and titratable acids were all lower than those of CK. Chu^50^ found that the application of organic fertilizer can increase TSS and TSS/TA of citrus. Luo et al.^51^ found that organic fertilizer can increase the Vc content of Feicheng peach fruits and reduce the titratable acid content. Organic fertilizer could continuously and slowly release fertilizer efficiency, which was conducive to synchronizing with the physiological needs of crops. It could effectively promote the coordinated and balanced nutrient metabolism of crops to ensure high yield and high quality of crops^52^.

Fertilizer application can be based on leaf synergistic efficiency and fruit quality as reference indicators^53^. Based on the above results, applying organic fertilizers can promote vegetative growth, reproductive growth, quality improvement and water utilization of jujube trees. Among them, the effect of SC fertilizer is relatively best, which can provide a theoretical basis and reference for scientific fertilization for the production of organic Pear-jujube in the hilly area of the Loess Plateau. However, further research is needed to determine the appropriate application rate of organic fertilizer, comprehensive effect of organic fertilizer application, and cumulative effect of long-term application of organic fertilizer and its impact on soil fertility.

## Conclusions

1. The application of soybean cake fertilizer (SC) had the most significant effect on the improvement of jujube hanging and leaf area; the application of organic fertilizer increased the LAI, canopy light interception density average, Pn and Gs, where the BM processed reach a maximum. The SWC of SC was significantly higher than that of CK and CF.
2. The application of organic fertilizer can significantly increase the fruit yield of Pear-jujube, and the yield of SC treatment is the highest.
3. Each organic fertilizer treatment significantly improved the fruit nutritional quality. TSS, TSS/TA, Vc and total flavonoid content of each treatment were higher than those of CK. The SC treatment had the highest water content, and the titratable value of SC acid content is minimal.

In the semi-arid area of northern Shaanxi, water and fertilizer are the main factors affecting Pear-jujube. From the perspective of fertilizer, the application of organic fertilizer in the loess hilly area of northern Shaanxi can effectively promote the growth and development of dwarf densely planted Pear-jujube, increase yield, and significantly improve fruit quality, among which cake fertilizer has the most significant effect. Compared with conventional fertilization, applying 5 kg of soybean cake fertilizer per plant can supply Pear-jujube trees all year round and need complete fertilizer. Therefore, the fertilizer should be applied at one time when the base is applied, and the required dosage should be reasonable, and the organic fertilizer must be fully decomposed.

## Supplementary Information

Supplementary Information.

## Data Availability

All data generated or analyzed during this study are included in this published article.
